# WHO GETS RESOURCES ACROSS THE LIFESPAN? A COORDINATED DATA ANALYSIS OF PERSONALITY AND SOCIOECONOMIC CONDITIONS

**DOI:** 10.1093/geroni/igad104.1648

**Published:** 2023-12-21

**Authors:** Stephen Antonoplis, Olivia Atherton, Eileen Graham, Daniel Mroczek

**Affiliations:** Northwestern University, Chicago, Illinois, United States; University of Houston, Houston, Texas, United States; Northwestern University, Chicago, Illinois, United States; Northwestern University, Chicago, Illinois, United States

## Abstract

From schooling in early life to employment, income, and generating wealth in adulthood, people must navigate socioeconomic conditions throughout their entire life. Personality traits have been shown to predict how people navigate socioeconomic conditions, and a number of open questions remain. In this project, we address several of these questions, including 1.) do stable, between-person trait levels or within-person fluctuations matter for socioeconomic outcomes?, 2.) do traits matter across the lifespan for socioeconomic outcomes or only in specific age periods?, and 3.) do periods of economic recession change the relationship between traits and socioeconomic outcomes? To answer these questions, we examine the prospective associations between personality traits and socioeconomic conditions across the lifespan, using eleven longitudinal datasets. Participants were born from 1880–2000 and aged 5–102; myriad ages and generations were present for all major U.S. military and economic events of the last 50 years. The samples were racially diverse, containing datasets focused on African American, Asian American, and Pacific Islander experiences, and several contemporary nationally representative datasets. Initial results show, for income for instance, that 1.) between-person levels, not within-person fluctuations, matter, and 2.) between-person levels matter across all ages. Openness to Experience, Conscientiousness, and Extraversion predicted earning a higher yearly income; Neuroticism predicted earning a lower yearly income. Effects sizes for traits were comparable to that for parental education. Our results shed light on how people navigate social structures across the lifespan.

